# Exploring the COVID-19 Pandemic as a Catalyst for Behavior Change Among Patient Health Record App Users in Taiwan: Development and Usability Study

**DOI:** 10.2196/33399

**Published:** 2022-01-06

**Authors:** Chinyang Henry Tseng, Ray-Jade Chen, Shang-Yu Tsai, Tsung-Ren Wu, Woei-Jiunn Tsaur, Hung-Wen Chiu, Cheng-Yi Yang, Yu-Sheng Lo

**Affiliations:** 1 Department of Computer Science and Information Engineering National Taipei University New Taipei Taiwan; 2 Department of Surgery School of Medicine, College of Medicine Taipei Medical University Taipei Taiwan; 3 Taipei Medical University Hospital Taipei Taiwan; 4 Graduate Institute of Biomedical Informatics College of Medical Science and Technology Taipei Medical University Taipei Taiwan; 5 Industrial Technology Research Institute Hsinchu Taiwan

**Keywords:** personal health records, COVID-19, My Health Bank, blockchain, public health

## Abstract

**Background:**

During the COVID-19 pandemic, personal health records (PHRs) have enabled patients to monitor and manage their medical data without visiting hospitals and, consequently, minimize their infection risk. Taiwan’s National Health Insurance Administration (NHIA) launched the My Health Bank (MHB) service, a national PHR system through which insured individuals to access their cross-hospital medical data. Furthermore, in 2019, the NHIA released the MHB software development kit (SDK), which enables development of mobile apps with which insured individuals can retrieve their MHB data. However, the NHIA MHB service has its limitations, and the participation rate among insured individuals is low.

**Objective:**

We aimed to integrate the MHB SDK with our developed blockchain-enabled PHR mobile app, which enables patients to access, store, and manage their cross-hospital PHR data. We also collected and analyzed the app’s log data to examine patients’ MHB use during the COVID-19 pandemic.

**Methods:**

We integrated our existing blockchain-enabled mobile app with the MHB SDK to enable NHIA MHB data retrieval. The app utilizes blockchain technology to encrypt the downloaded NHIA MHB data. Existing and new indexes can be synchronized between the app and blockchain nodes, and high security can be achieved for PHR management. Finally, we analyzed the app’s access logs to compare patients’ activities during high and low COVID-19 infection periods.

**Results:**

We successfully integrated the MHB SDK into our mobile app, thereby enabling patients to retrieve their cross-hospital medical data, particularly those related to COVID-19 rapid and polymerase chain reaction testing and vaccination information and progress. We retrospectively collected the app’s log data for the period of July 2019 to June 2021. From January 2020, the preliminary results revealed a steady increase in the number of people who applied to create a blockchain account for access to their medical data and the number of app subscribers among patients who visited the outpatient department (OPD) and emergency department (ED). Notably, for patients who visited the OPD and ED, the peak proportions with respect to the use of the app for OPD and ED notes and laboratory test results also increased year by year. The highest proportions were 52.40% for ED notes in June 2021, 88.10% for ED laboratory test reports in May 2021, 34.61% for OPD notes in June 2021, and 41.87% for OPD laboratory test reports in June 2021. These peaks coincided with Taiwan’s local COVID-19 outbreak lasting from May to June 2021.

**Conclusions:**

This study developed a blockchain-enabled mobile app, which can periodically retrieve and integrate PHRs from the NHIA MHB's cross-hospital data and the investigated hospital's self-pay medical data. Analysis of users’ access logs revealed that the COVID-19 pandemic substantially increased individuals’ use of PHRs and their health awareness with respect to COVID-19 prevention.

## Introduction

Electronic medical records (EMRs) have enabled the digital transformation of health facilities worldwide [1-4]. With the widespread development and use of wearable devices, electronic health records (EHRs) can be generated and recorded effectively, and their adoption outside of the hospitals has increased rapidly [5-9]. Consequently, health data are expected to become more personalized and self-controlled after they are converted into personal health records (PHRs) [8-11]. With PHRs, patients can access, control, and track their health data and minimize hospital visits. Many countries have implemented national PHR systems that empower people to participate actively in their care and choose between opt-in and opt-out models [12-16]. However, despite the emerging trend of using PHRs, the PHR adoption rate remains low because of multiple challenges and barriers, including interoperability challenges relating to interfacility EMR and EHR access, implementation costs, barriers imposed by health care data security and privacy regulations (such as General Data Protection Regulation), and the assessment of relevant benefits for patients, health care providers, and health insurance institutes [7,8,17,18].

In 2014, Taiwan’s National Health Insurance Administration (NHIA) launched the My Health Bank (MHB) 1.0 service [19]. Similar to other nation-based PHR systems [14-16], the MHB service provides a personal health account for each insured individual. Notably, the MHB continually collects insured individuals’ medical data pertaining to outpatient visits, hospitalizations, dental services, traditional Chinese medicine outpatient visits, allergies, pathological test reports, X-ray, computed tomography, and magnetic resonance imaging examination results, discharge summaries, advance directives relating to organ donation and palliative care, vaccinations, and preventive care [20]. When an insured individual wants to retrieve his/her medical data, he/she can visit the MHB website, insert his/her National Health Insurance (NHI) smart card into a card reader, key in the corresponding password for the card, and download his/her MHB data as an XML, HTML, or JSON file after completing a verification process. In 2016, an updated version of the MHB, MHB 2.0, was launched. The new MHB incorporates an advanced encryption method that enables an insured individual to use his/her NHI smart card number and password to access his/her MHB data without having to use the physical card. In 2019, the NHIA released the MHB software development kit (SDK), which can be used to develop a mobile app for retrieving MHB data through an identity verification process.

The MHB SDK can attract more health care providers and startups to create innovative mobile apps in the health industry and encourage them to invest their resources into realizing market opportunities. It can also drive more users to engage in the control and management of their health data. However, the MHB SDK has several limitations that must be addressed. First, only the 3 most recent years of an individual’s data are stored in the MHB. Therefore, a person cannot retrieve older medical data [16]. Second, the private medical or health data of self-pay patients, such as health checkup reports, are not stored in the MHB. Third, the personal MHB that an individual has downloaded is saved and stored in his/her mobile device. Hence, a data protection mechanism is required for such data given their sensitivity and confidentiality.

Because of the weak gatekeeper role of Taiwan’s NHI system, patients can freely seek clinical services without referral whenever they feel uncomfortable [21]. Thus, they may not be aware of the benefits of using their PHRs. Moreover, NHI-insured individuals’ participation in the NHIA MHB service is voluntary and not incentivized, and the corresponding participation rate thus remains low [19]. During the COVID-19 pandemic, the Taiwan government introduced a Name-Based Mask Distribution system that is linked to the NHIA MHB service. This system is a rationing system for face masks, which allows the public to purchase masks at a convenience store. Thus, the previously poor participation rates of the NHIA MHB service increased substantially during the COVID-19 pandemic [22]. In other words, people may have become aware of their medical data through the NHIA MHB service when they visited a hospital during the COVID-19 pandemic. Therefore, the COVID-19 pandemic may provide an opportunity for improving PHR use and accelerating the digital transformation of Taiwan’s health care system.

In our previous study, we adopted the Go Ethereum version 1.7.3-stable to construct the iWellChain Framework, which is a permissioned consortium blockchain network with trusted parties to ensure consensus by proof of authority. Thus, the framework can limit participants who transact on the blockchain and define users who can serve the network by writing new blocks into the chain. In this way, the iWellChain Framework can assist the cooperative health care parties to establish their blockchain environment efficiently and further acquire the patient's EMRs and EHRs in accordance with the patient’s signed Ethereum-based smart contracts. Accordingly, the framework could help health care parties to reduce their implementation costs and difficulties associated with the hospital’s legacy information technology systems and security features surrounding data access. Moreover, the iWellChain Framework provides a better secure data protection because there is no centralized structure for a malicious user to target, as the patients’ EMRs and EHRs are stored in numerous copies on different blockchain nodes. With the iWellChain Framework, we also developed a blockchain-enabled mobile app, the iWellChain app, as a PHR tool for patients to acquire their EMRs and EHRs from the investigated hospital and cooperative clinics [23]. Launched on September 16, 2018, the iWellChain app is compatible with both iOS and Android devices. Thus, when a family doctor or specialist is interested in the referral patient’s EMRs and EHRs, the patient can use the iWellChain app to authorize and set the approved period for the physician to access the EMRs and EHRs. However, it was a pilot study, and the limitations of the iWellChain app were that the patient could not use it to retrieve the patient’s EMRs and EHRs from other health care facilities. Accordingly, our practical experience has indicated that iWellChain app is less popular than expected.

In this study, we aimed to integrate the MHB SDK with our developed blockchain-enabled PHR mobile app, which enables patients to access, store, and manage their cross-hospital PHR data. We also collected and analyzed the app’s log data to examine patients’ MHB use during the COVID-19 pandemic. This study involved the launch of an updated version of the iWellChain app in July 2019. The updated version incorporates the MHB SDK. Therefore, patients can regularly use it to retrieve, store, and track cross-hospital MHB data. Consequently, the iWellChain app addresses time restrictions related to the MHB data retention period. Additionally, the iWellChain app incorporates blockchain technology. Hence, a user can employ a public key to encrypt downloaded MHB data and ensure that their personal health data are secure. Furthermore, to encourage more individuals to use the iWellChain app, the investigated hospital released patients’ self-pay medical data and health checkup reports, which are not available in the MHB database. Finally, to enhance our understanding of patients’ PHR use during the COVID-19 pandemic, we analyzed the access logs of iWellChain app users to examine patients’ activities during a 24-month study period.

## Methods

### Settings

This study was conducted at the Taipei Medical University Hospital (TMUH)—a teaching hospital with almost 800 beds and a good information infrastructure. In 2018, this hospital released five types of EMR and EHR that patients can access through the iWellChain app: outpatient department (OPD) notes, OPD laboratory test reports, discharge notes, pathology reports, and health check reports. An updated version of the iWellChain app was integrated with the MHB SDK and released in July 2019. This version enables patient access to emergency department (ED) notes and ED laboratory test reports.

### Integration Workflow of the iWellChain App and MHB SDK

**Figure 1:**
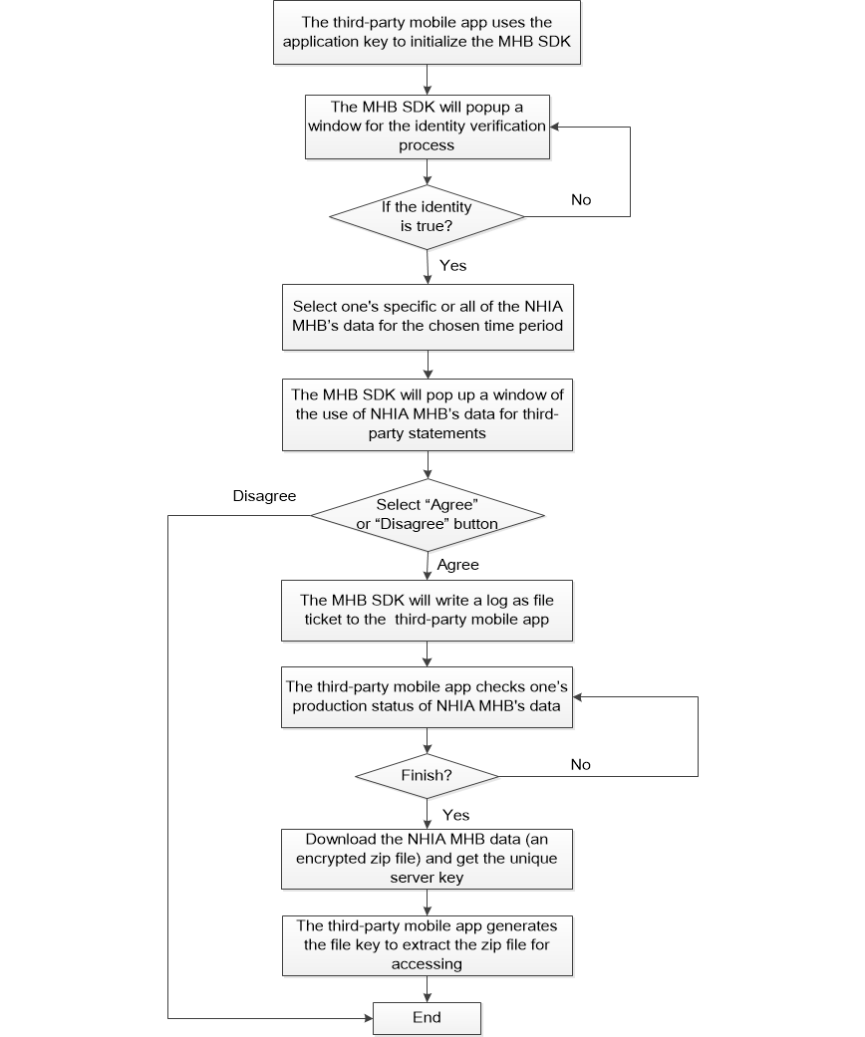
Integration workflow of the My Health Bank software development kit and mobile app. MHB: My Health Bank, NHIA: National Health Insurance Administration, SDK: software development kit.

**Figure 2:**
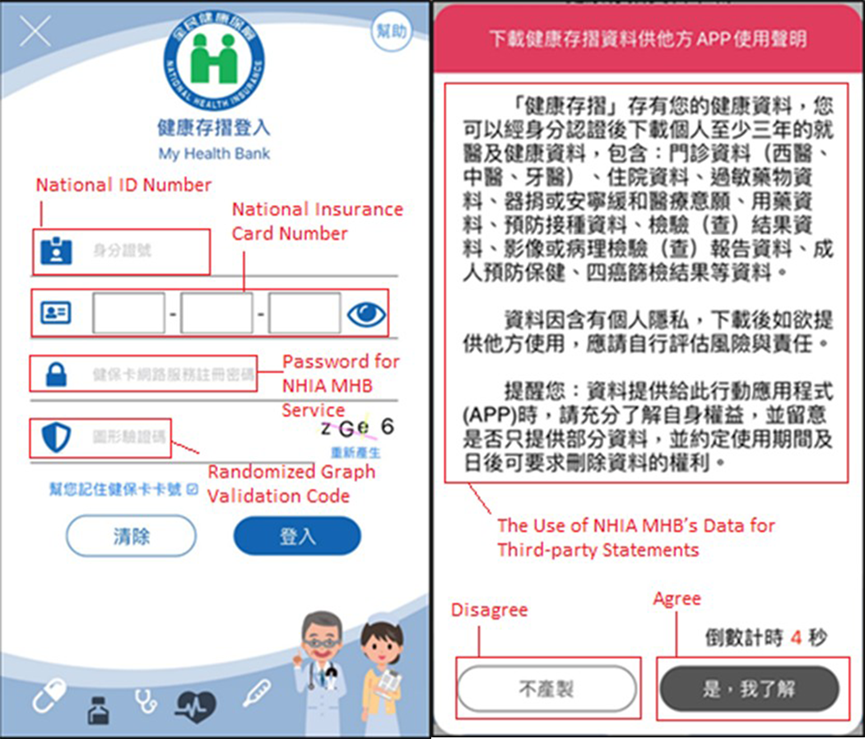
Identity verification process of the My Health Bank software development kit for accessing My Health Bank services (left panel); third-party statements relating to use of My Health Bank data (right panel).

The MHB SDK was used to develop a mobile app that enables users to access their NHIA MHB data by completing an identity verification process. Figure 1 presents the integration workflow of the MHB SDK and mobile app. First, the mobile app user employs an application key acquired from and approved by the NHIA to initialize the MHB SDK. Thereafter, the mobile app invokes the MHB SDK to sign into the user’s NHIA MHB account, and the user then completes an identity verification process that requires the user’s national ID number, national insurance card number, password for the NHIA MHB service, and a randomized graph validation code (left panel of Figure 2). When the user’s identity has been verified, he/she can select specific MHB data or all NHIA MHB data recorded within a specific time period. Next, the MHB SDK displays a window containing third-party statements relating to the use of NHIA MHB data (right panel of Figure 2). After the user agrees to the stated terms and conditions, the MHB SDK retrieves the user’s NHIA MHB data in accordance with the set conditions and writes and stores the data to a log as a file ticket in the mobile app. Second, through the MHB SDK, the mobile app can use the file ticket to check the applicant’s production status with respect to the NHIA MHB data on the NHIA server side. If the server process has not yet been completed, the MHB SDK then sends the mobile app a message stating that “No files can be downloaded.” After the server process is complete, the mobile application then requests the applicant’s MHB data for download, and the MHB SDK sends the message “The files are fully downloaded” and a unique server key to the mobile app. The NHIA MHB data are downloaded as a zip file that is encrypted using the Public-Key Cryptography Standards #5 password-based cryptography method defined in RFC 2898 [24]. Third, the mobile app uses the received application key as the password and the unique server key as the salt to generate a file key. Next, the mobile app uses the file key to extract the zip file and access the applicant’s MHB data.

### Analysis of NHIA MHB Data

**Figure 3:**
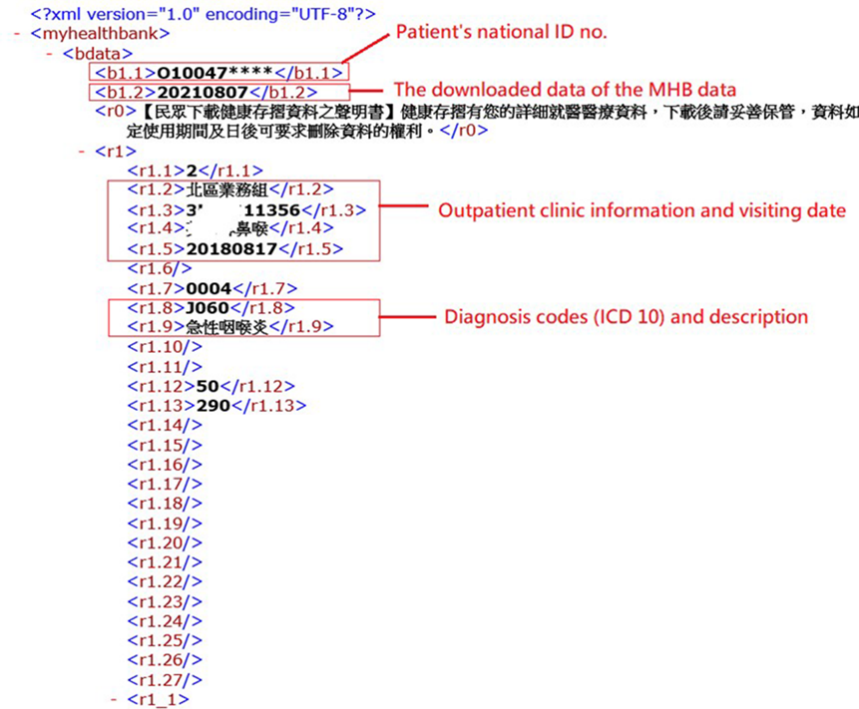
Downloaded My Health Bank data in XML format.

The content of the downloaded NHIA MHB data is presented in a tagged format, such as XML or JASON format. Each tag contains a name, value, and the corresponding medical data. Table 1 presents the tag name and its corresponding medical data. Figure 3 presents the NHIA MHB data in the XML format. For example, the tag name “r1” represents outpatient clinic data, which include clinic information, the dates of visits, diagnosis codes, and descriptions. Accordingly, the iWellChain app extracts a patient's relevant medical data from the NHIA MHB data for further processing.

**Table 1:** Tag codes and values of National Health Insurance Administration My Health Bank data.

Name	Value
bdata	Patient information
r1	Outpatient clinic data
r2	Admission data
r3	Dental clinic data
r4	Allergy data
r5	Organ donation or palliative medicine
r6	Vaccination data
r7	Laboratory data
r8	Imaging or pathological examination reports
r9	Chinese medicine data
r10	Health and preventive care

### Data Processing and Synchronization Between iWellChain App and NHIA MHB Data

**Figure 4:**
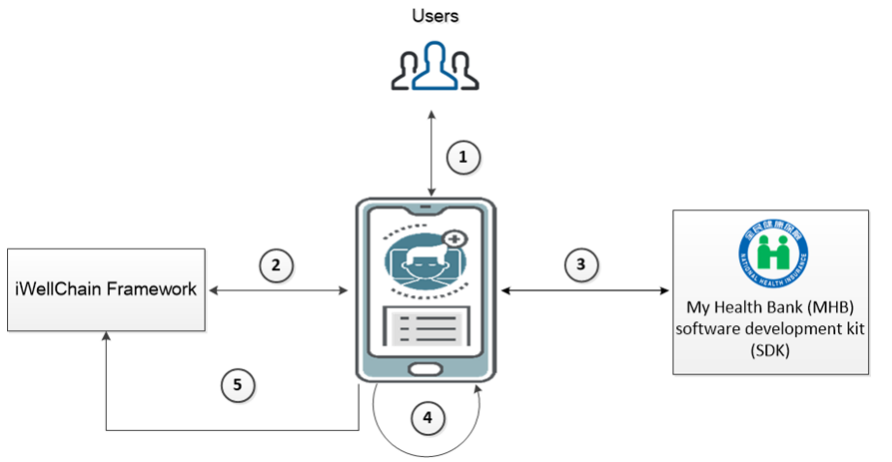
Interactions with the iWellChain app and My Health Bank data through the iWellChain Framework.

In our previous study, we constructed the iWellChain Framework—a permissioned consortium blockchain that utilizes trusted parties to ensure consensus by proof of authority [23]. Figure 4 describes the 5 steps of data processing and synchronization achieved through the iWellChain Framework between the iWellChain app and NHIA MHB data.

After a patient has created his/her blockchain account, he/she can use his/her national ID and application password and the password for the private key to sign into the iWellChain app.

Next, the iWellChain app synchronizes his/her Ethereum blockchain ledger through the iWellChain Framework. Thus, the patient can quickly obtain EMR and EHR indexes and further acquire the physical EMR and EHR files of these indexes using the iWellChain app.

After a patient has successfully signed into the NHIA MHB, his/her NHIA MHB data are downloaded. Once the NHIA MHB data are fully downloaded, the iWellChain app decrypts these data and stores them in the mobile app sandbox.

The iWellChain app then analyzes each of the patient’s NHIA MHB data records to verify that these records were not previously acquired or did not originate from other health facilities.

For each record that has been verified as a new record, the iWellChain app synchronizes with the iWellChain Framework to request the patient’s public key, which is then used to encrypt the record’s path. Finally, the iWellChain app registers the record's information and its encrypted path in the iWellChain Framework.

### Trends in the COVID-19 Pandemic in Taiwan

**Figure 5:**
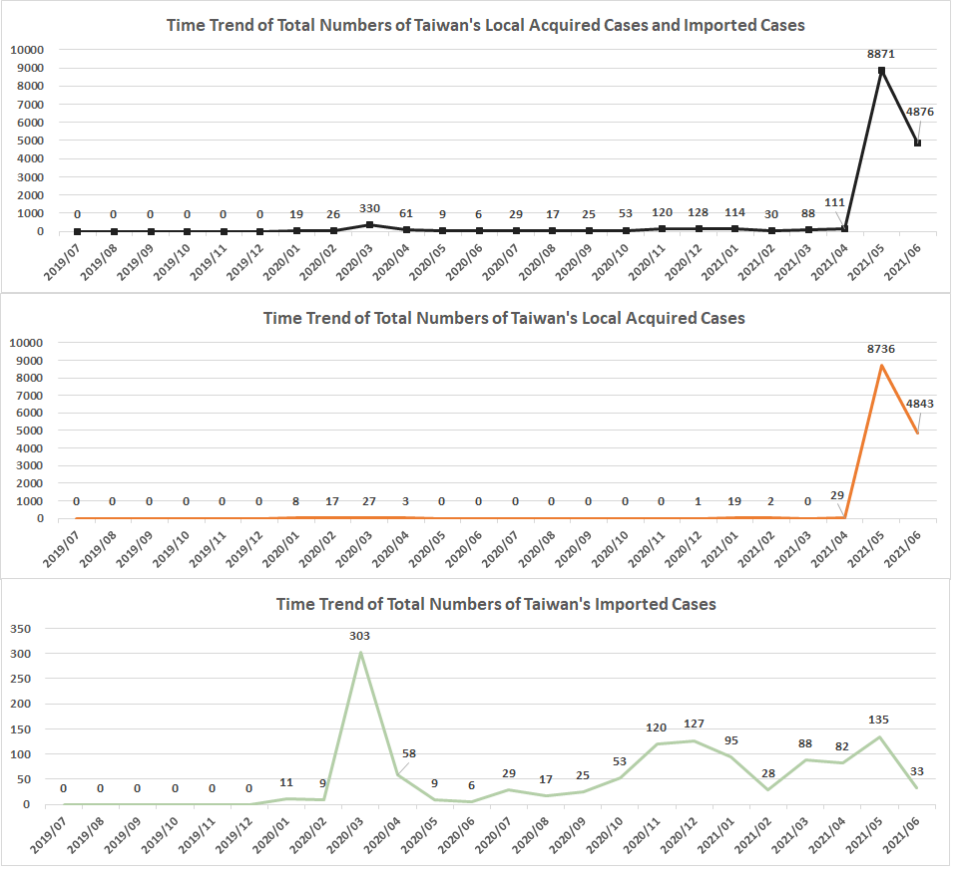
Taiwan’s locally acquired and imported case trends.

According to Taiwan’s Central Epidemic Command Center (CECC) reports, confirmed COVID-19 cases can be classified as locally acquired cases (LACs) and imported cases (ICs). Figure 5 indicates that both LACs and ICs started arising in January 2020. From January to April 2020, both LACs and ICs were reported: 19 (8 LACs, 11 ICs), 26 (17 LACs, 9 ICs), 330 (27 LACs, 303 ICs), and 61 (3 LACs, 58 ICs) cases were reported in January, February, March, and April 2020, respectively. During the subsequent 7-month period (May 2020 to November 2020), no LACs were reported. However, the number of ICs reported fluctuated from month to month. During the next 3-month period (December 2020 to February 2021), both LACs and ICs were reported: 128 (1 LAC, 127 ICs), 114 (19 LACs, 95 ICs), and 30 (2 LACs, 28 ICs) cases were reported in December 2020, January 2021, and February 2021, respectively. In March 2021, 0 LACs and 88 ICs were reported. From April to June 2021, the number of LACs reported increased substantially: 29 LACs and 82 ICs, 8,734 LACs and 135 ICs, and 4,785 LACs and 33 ICs were reported in April, May, and June 2021, respectively. Accordingly, the CECC raised the COVID-19 alert level to level 3 nationwide and maintained it from May 19 to July 26, 2021. Per COVID-19 level 3 alert guidelines, places of business and public venues were recommended to stop operating (excluding essential services, law enforcement, medical treatment facilities, and government offices). However, for places that remained open, strict mask-wearing and social-distancing protocols were enforced [25].

### Log Data Collection and Analysis of the iWellChain App

Because of the COVID-19 pandemic, the investigated hospital had to adhere to CECC guidelines regarding the reservation of general wards for the influx of patients with COVID-19. Accordingly, in this study, we only included patients attending the OPD and ED of TMUH and used the iWellChain app. We retrospectively collected patients’ iWellChain app access logs over a 24-month period (before and during the COVID-19 pandemic) from July 2019 to June 2021. Accordingly, an access log database was created, and information on the actions that the patients performed through the iWellChain app was collected. We integrated Google Analytics for Firebase into the iWellChain app to capture patients’ click data. Once the data had been captured and stored, the access log could be linked to the Google Colaboratory [26]—a web-based and free Python development platform for further analysis and reporting. The Joint Institutional Review Board of Taipei Medical University and TMUH approved this study.

## Results

### PHR Features of the iWellChain App

**Figure 6:**
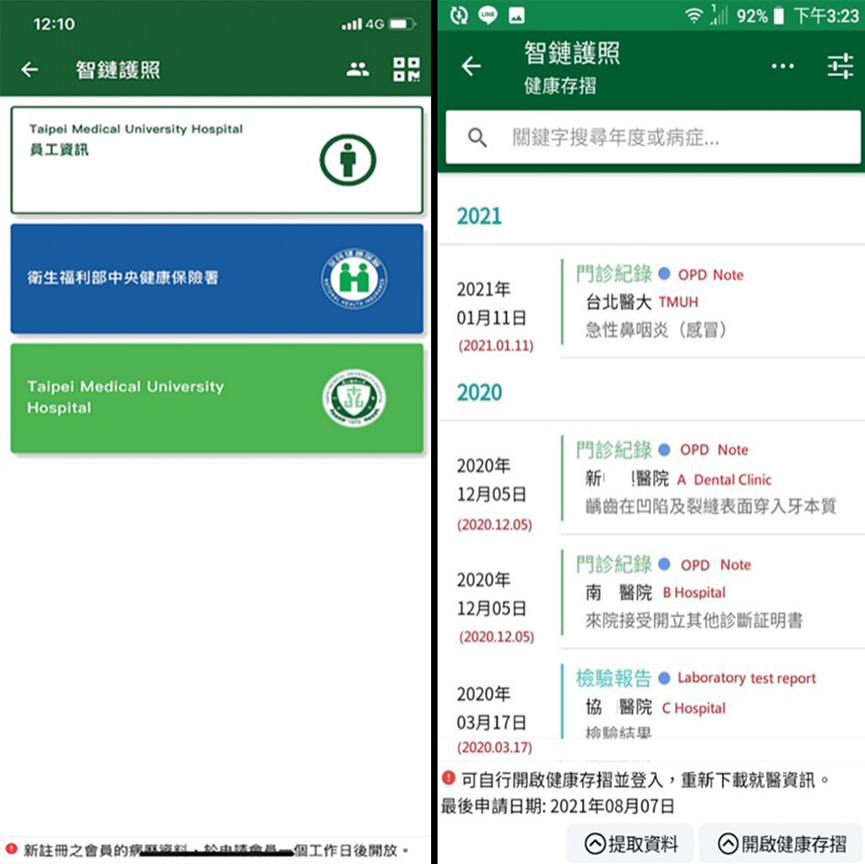
Patient's subscribed personal health record services in the iWellChain app (left panel) and medical data sorted by visit date from latest to oldest.

**Figure 7:**
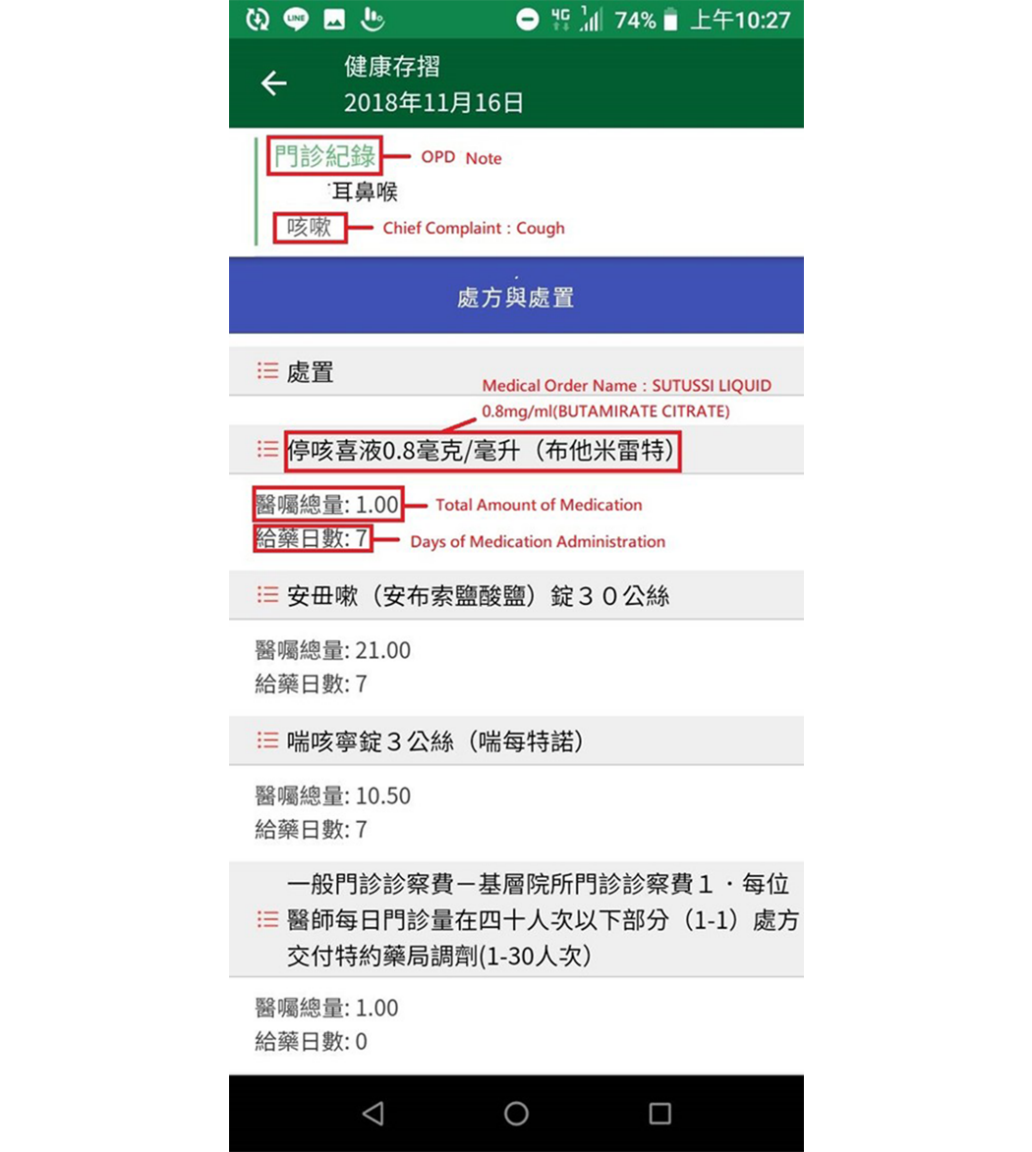
Patient’s outpatient department notes and corresponding prescription information.

When a patient signs into his/her blockchain account, the iWellChain app displays his/her subscribed PHR services per the signed Ethereum-based smart contracts. Figure 6 (left panel) presents an example of a patient who subscribed to three PHR services: the employee card service (top section), NHIA MHB card service (middle section), and TMUH member card service (bottom section). The employee card service is designed to enable employees to access their annual regular checkup reports. This service can also be used to establish an employee’s personal health profile for long-term health tracking and management. The NHIA MHB card service can activate the MHB SDK to retrieve an individual’s MHB data after he/she has completed the identity verification process. The TMUH member card service provides a single window in which the patient can acquire and integrate EMR, EHR, and NHIA MHB data as the PHR data. Figure 6 (right panel) presents the timeline of a patient's medical data that were sorted in accordance with his/her visit dates (from latest to oldest). For example, the patient had three OPD notes and one laboratory test report. The first OPD note was issued by the investigated hospital on January 11, 2021. The next two OPD notes and one laboratory test report were retrieved from the patient’s NHIA MHB data. The two OPD notes were issued by dental clinic A and hospital B on December 5, 2020, and the laboratory test report was issued by hospital C on March 17, 2020. The patient could also select the specific medical data to view more details. Figure 7 presents the patient’s prescription information, which includes names of medical orders, total medication dosages, and days of medication administration.

### Study Population

We collected 24 months of data (July 1, 2019, to June 30, 2021) from the investigated hospital. During this 2-year period, the total number of patients who visited the OPD and ED was 1,405,316, and 92,184, respectively. From July 1, 2019, to December 30, 2019, the proportion of patients who visited the ED and OPD who subscribed to the iWellChain app ranged from 0.29% to 1.67% and from 3.09% to 5.36%, respectively. In 2020, the proportion of patients who visited the ED and OPD who subscribed to the iWellChain app ranged from 1.41% to 2.54% and from 5.29% to 6.25%, respectively. Finally, from January 1, 2021, to June 30, 2021, the proportion of patients who visited the ED and OPD who subscribed to the iWellChain app ranged from 1.95% to 8.20% and from 5.58% to 7.49%, respectively. Therefore, the iWellChain subscription trend for patients who visited the OPD or ED was a steady increase during the investigated period. The descriptive statistics for the study population are presented in Table 2.

**Table 2:** Proportion of patients who visited the outpatient department and emergency department and who subscribed to the mobile app between July 2019 and June 2021.

Month#		ED^a^ patients, n	ED patients subscribed to the app, n (%)	OPD^b^ patients, n	OPD patient subscribed to the app, n (%)
**2019**					
	7	4199	12 (0.29)	65,152	2011 (3.09)
	8	4194	22 (0.52)	62,841	2178 (3.47)
	9	4266	27 (0.63)	61,108	2354 (3.85)
	10	4250	17 (0.4)	64,454	2544 (3.95)
	11	3877	32 (0.83)	64,080	2676 (4.18)
	12	4187	70 (1.67)	63,709	3413 (5.36)
**2020**					
	1	4980	89 (1.79)	59,506	3150 (5.29)
	2	3421	71 (2.08)	53,918	2919 (5.41)
	3	3226	68 (2.11)	48,970	3052 (6.23)
	4	2842	51 (1.79)	47,116	2833 (6.01)
	5	3186	45 (1.41)	54,119	3185 (5.89)
	6	3427	76 (2.22)	54,100	3308 (6.11)
	7	3549	90 (2.54)	63,144	3829 (6.06)
	8	3577	81 (2.26)	61,902	3607 (5.83)
	9	3647	72 (1.97)	63,712	3680 (5.78)
	10	4040	71 (1.76)	65,443	3667 (5.6)
	11	3788	74 (1.95)	61,820	3707 (6)
	12	3546	74 (2.09)	63,752	3986 (6.25)
**2021**					
	1	3835	79 (2.06)	59,872	3530 (5.9)
	2	3602	89 (2.47)	52,761	2942 (5.58)
	3	3995	78 (1.95)	65,574	4083 (6.23)
	4	3829	82 (2.14)	64,043	3812 (5.95)
	5	5025	412 (8.2)	46,908	3394 (7.24)
	6	3696	208 (5.63)	37,312	2794 (7.49)

^a^ED: emergency department.

^b^OPD: outpatient department.

### New Blockchain Account Applicants

**Figure 8:**
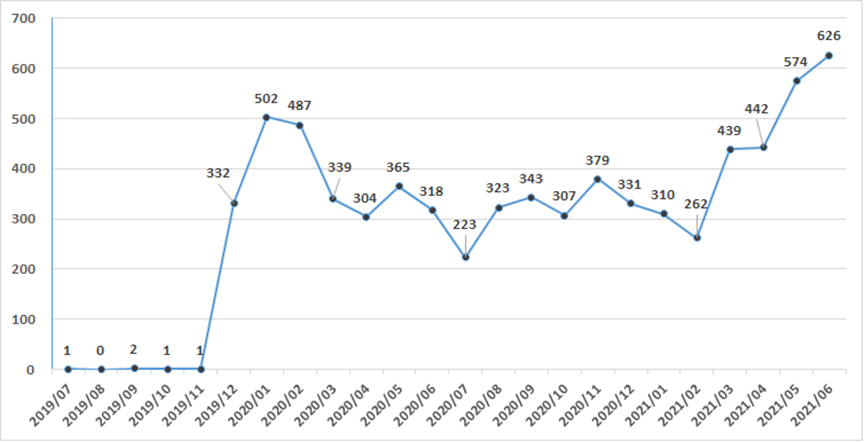
Numbers of new applicants of the blockchain accounts.

The iWellChain app has several basic functions (ie, an appointment service, inquiring for a doctor’s information, and a payment service); however, patients have to apply for and register web-based blockchain accounts to acquire their medical data. Figure 8 presents the trend in the numbers of new applicants. During the study period, the total number of patients who applied for a blockchain account was 7211. From July to December 2019, only a few new people applied; specifically, the number of new applicants was 1, 0, 2, 1, 1, and 332 in July, August, September, October, November, and December 2019, respectively. Since January 2020, the number of new applicants increased substantially, and the number of applicants was 6874 (95.33%) from January 2020 to June 2021. The number of applicants peaked at 626 in June 2021; the second (574) and third (502) highest numbers were observed in May 2021 and January 2020, respectively. The peak in the number of new applicants coincided with the onset of COVID-19 and outbreak of LACs in Taiwan.

### Analysis of Patients’ Access Log Data for Medical Data Acquisition

Through the access log data, we further examined the investigated hospital’s data set of patients who visited the OPD and ED, with data including OPD notes, OPD laboratory test results, ED notes, and ED laboratory test reports. The content of the OPD and ED notes include OPD or ED visit dates, chief complaints, diagnosis codes and descriptions, and prescription information.

**Figure 9:**
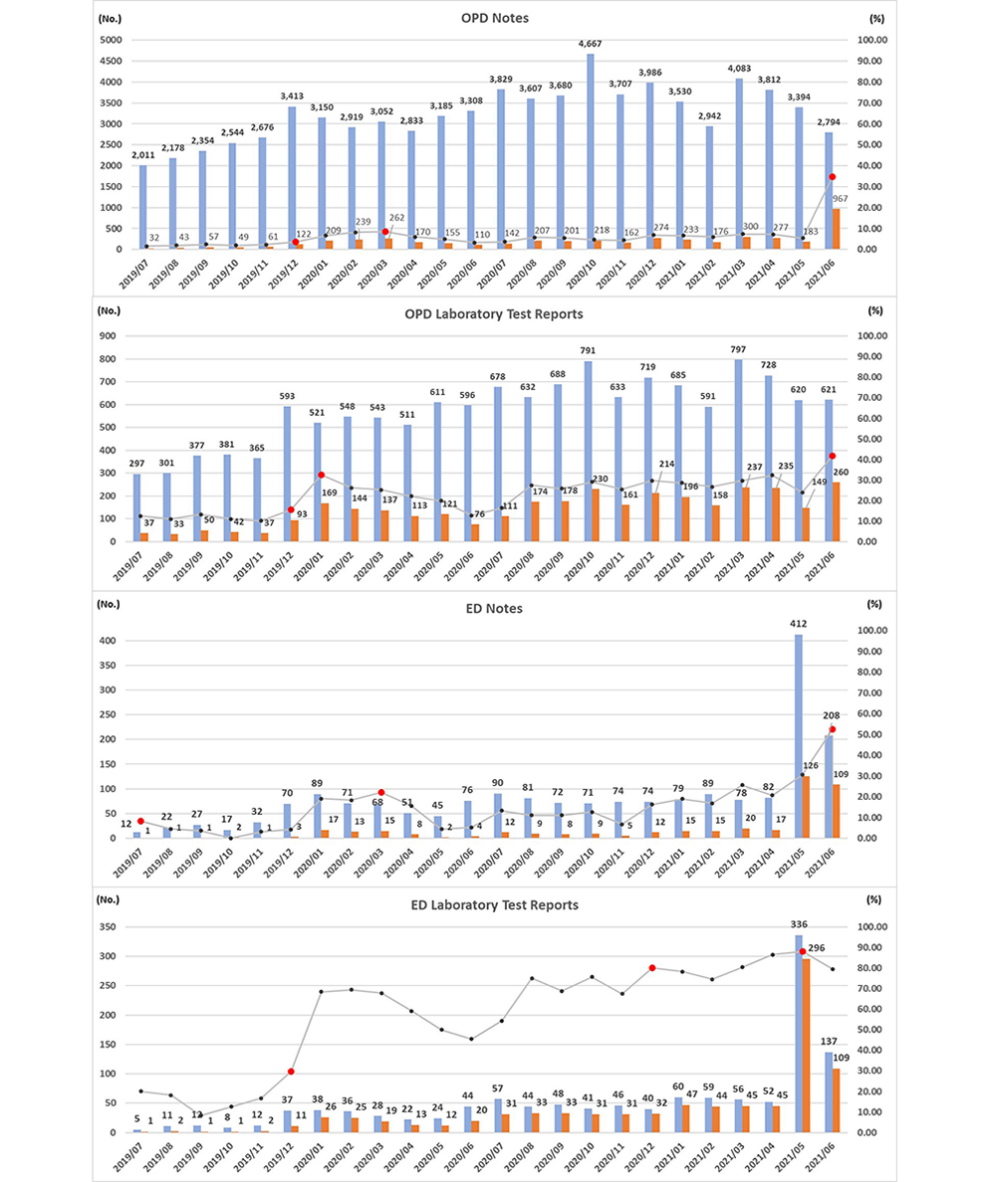
Analysis of patients’ access log data with respect to acquisition of outpatient department (OPD) notes, OPD laboratory test results, emergency department (ED) notes, and ED laboratory test reports through the iWellChain app.

In Figure 9, the left axis indicates that the numbers of patients visiting the OPD and ED who subscribed to the iWellChain app and had visited the hospital (A) and the numbers of patients who used the iWellChain app to acquire the corresponding OPD notes, OPD laboratory test results, ED notes, and ED laboratory test reports (B). The right axis represents the value obtained by dividing value (B) by value (A) (B/A). The number (A) and proportion (B/A) of patients who visited the OPD and ED for each month varied depending on the type of medical data.

During the 2-year study period, subscribers who retrieved OPD or ED notes and laboratory test reports accounted for the highest proportion of patients who visited the OPD or ED in May and June 2021. These peak proportions were 52.40%, 88.10%, 70.30%, 34.61%, 41.87%, and 59.85% for OPD notes (June 2021), OPD laboratory test results (June 2021), ED notes (June 2021), and ED laboratory test reports (May 2021), respectively.

From July 2019 to December 2019, only a few patients visiting the OPD or ED subscribed to the iWellChain app and used it to acquire their OPD or ED notes and laboratory test reports (Figure 9). For example, 2011, 2178, 2354, 2676, 2715, and 3414 patients who visited the OPD subscribed to the application in July, August, September, October, November, and December 2019, respectively. Among these patients, only 32 (1.59%), 43 (1.97%), 57 (2.42%), 49 (1.93%), 61 (2.28%), and 122 (3.57%) used the iWellChain app to acquire their OPD notes in July to December 2019, respectively. Furthermore, only some patients who visited the OPD underwent laboratory tests. Among the patients who visited the OPD who subscribed to the app in July, August, September, October, November, and December 2019, only 297, 301, 377, 381, 365, and 593, respectively, underwent laboratory tests. Among them, only 37 (12.46%), 33 (10.96%), 50 (13.26%), 42 (11.02%), 37 (10.14%), and 93 (15.68%) patients used the iWellChain app to acquire their OPD laboratory test reports, respectively.

In 2019, for each type of medical data, the peak proportions were thus 8.33% for ED notes (July 2019), 29.73% for ED laboratory test reports (December 2019), 3.57% for OPD notes (December 2019), and 15.68% for OPD laboratory test reports (December 2019). In 2020, the peak proportions for each type of medical data were all higher than those observed in 2019. Moreover, from January to June 2021, the peak proportions for each type of medical data and report were all higher than those observed in 2019 and 2020.

## Discussion

### Principal Findings

Taiwan’s NHI system is a single-payer system that was introduced in 1995; by 2010, it was already providing universal and mandatory insurance coverage for almost all of Taiwan’s population (99.5%) [27,28]. The continual accumulation of NHI MHB data provides a favorable opportunity for using the data as a catalyst to improve PHR use through the MHB SDK. Utilization of the MHB SDK to implement a PHR app is a promising implementation method because of the low implementation cost, high data quality, and availability without participation bias [28].

In this study, we successfully integrated the MHB SDK into our blockchain-enabled mobile app to enable use by patients. The iWellChain app’s implementation has three advantages. First, iWellChain app can help patients periodically acquire their own cross-hospital EMRs and avoid the time restrictions relating to the NHIA MHB data retention period. Second, to protect patients’ privacy and data security, the downloaded NHIA MHB data are stored on the patients’ mobile devices without moving these data to or depositing them in other storage or cloud-based spaces. With our method, patients feel more secure in using the iWellChain app for PHR data management. Third, the investigated hospital also released self-pay patients’ EMRs and EHRs (eg, health checkup reports) to complement the deficiencies of NHIA MHB data. However, under the Taiwan NHI system, people could easily obtain in-person clinic/hospital medical consultations with relatively low costs. Consequently, people would rather go to the hospital or clinic than use such the PHR mobile app to acquire their medical or health information. A previous study indicated the NHI-insured individuals’ participation rate in the NHIA MHB service was low [19]. Moreover, in this study, although the updated iWellChain app combined with the MHB SDK launched, it appeared not to attract more patients to use it for acquiring their PHRs in the beginning period (from July 2019 to December 2019). Accordingly, providing effective PHR mobile apps might be not the critical factor to increase the app users.

Before May 2021, Taiwan only had a few LACs (Figure 5). Thus, before May 2021, the willingness of Taiwanese people to be vaccinated was low, widespread screening—such as COVID-19 polymerase chain reaction (PCR) or rapid tests—was not urgently required, and few people used PHR apps. In May 2021, a COVID-19 outbreak occurred in tea houses located in Taipei City’s Wanhua District [29]. This outbreak quickly led to the announcement of a nationwide level 3 COVID-19 alert. Accordingly, the CECC established rapid testing sites in multiple cities, with the focus being Taipei City and New Taipei City. When an individual feels uncomfortable, he/she can go to a rapid testing site or a hospital to undergo COVID-19 rapid or PCR testing. After the test result has been released, the rapid testing site or hospital uploads the result to the NHIA database, which then synchronizes with the database used by the Taiwan Centers for Disease Control (CDC). However, the heavy load of mass screenings meant that individuals might have to wait between 3 and 6 days before they could obtain their test result and report on the CDC’s website or NHIA MHB's database. Concurrently, the trends observed in the investigated hospital (located in Taipei City) reflected the general trend in Taiwan’s COVID-19 outbreak. Compared with the government’s COVID-19 test reports, the iWellChain app reported the results of COVID-19 rapid and PCR tests within 1 day for patients who visited the OPD or ED of the investigated hospital. Figure 8 indicates that more people applied to create a blockchain account in May and June 2021 relative to other months. Table 2 indicates that the proportion of patients who visited the ED and OPD who subscribed to the app peaked at 8.20% (May 2021) and 7.49% (June 2021), respectively; among the aforementioned patients, the proportions who used the app to acquire their ED and OPD notes (peaks in May 2021) and laboratory test reports (ED peak in May 2021; OPD peak in June 2021) also peaked during the same period. These findings suggest that the COVID-19 pandemic led patients to pay more attention to their medical data. They also reflect a substantial increase in patients’ use of the PHR app in response to Taiwan’s local COVID-19 outbreak.

Under Taiwan’s NHI system, insured individuals’ medical data are routinely collected through a network with well-developed infrastructure. Although Taiwan has been affected by the COVID-19 outbreak, the CECC could effectively coordinate with all levels of health care facilities to control and manage epidemic information in response to the disease’s spread (eg, establishment of a rapid testing station in a hotspot or contact tracing) by implementing the existing infectious disease notification method. Taiwan lifted the Level 3 alert on July 27, 2021, and only a few LACs were reported in August 2021 [30]. Furthermore, the NHIA MHB database is being continually expanded with respect to vaccination information and progress. Similar to the bundling of the NHIA MHB service with mask purchase services, our investigated hospital provided the public with a web-based service for booking an appointment for a leftover vaccine through the iWellChain app [31]. This model could attract more users to use our iWellChain app.

### Conclusions

In a patient-centered model, EMRs and EHRs belong to the individual patient. Furthermore, Taiwan’s NHIA MHB database offers a robust foundation for PHR development. This study provides a blockchain-enabled mobile app that can periodically retrieve and integrate cross-hospital PHRs from the NHIA MHB database and the investigated hospital’s self-pay medical data and provide secure data protection through blockchain technology. The user access log analysis indicates that the COVID-19 pandemic has had a substantial effect on the app’s use, increasing individuals’ PHR use and health awareness regarding COVID-19 prevention. However, compared with the investigated hospital’s total number of patients who visited the OPD and ED, the number of app users remains low. Therefore, use of the iWellChain app for PHR acquisition can be further improved.

## Abbreviations

CDC: Centers for Disease Control
CECC: Central Epidemic Command Center
ED: emergency department
EHR: electronic health record
EMR: electronic medical record
IC: imported case
LAC: locally acquired case
MHB: My Health Bank
NHI: National Health Insurance
NHIA: National Health Insurance Administration
OPD: outpatient department
PCR: polymerase chain reaction
PHR: personal health record
SDK: software development kit
TMUH: Taipei Medical University Hospital
